# Personal risk perceptions for Alzheimer’s disease and dementia among older adults may not be grounded in knowledge

**DOI:** 10.1002/alz.091084

**Published:** 2025-01-09

**Authors:** Claudia Jacova, Frank Robertson, Jacklyn Gehling, Hannah Marx, Ravneet K. Dhaliwal, Steven Harris

**Affiliations:** ^1^ Pacific University, Hillsboro, OR USA

## Abstract

**Background:**

Public interest in brain health has reached unprecedented levels, yet research on AD/dementia literacy has continued to reveal gaps and misconceptions, especially among those with lower education. The public’s knowledge has often been characterized as particularly weak around AD/dementia risk factors. Here we were interested in whether personal risk perceptions among dementia‐free community‐dwelling older adults are evidence‐based. We investigated whether risk endorsement was associated with the expected personal characteristics, for example increased endorsement of age‐related risk with older age or of diabetes‐related risk with poorer health.

**Method:**

We recruited 279 community‐dwelling participants aged ≥60 years, free of neurocognitive diagnoses, and located within the United States (M age = 73.8, range 60‐96; M education (years) = 14.9, range 10‐20; 52% women). Participants completed an online survey through Qualtrics’ research panel teams where they indicated the factor/s (select all that apply) that contributed to their own risk for AD/dementia including demographic and modifiable factors. They also rated their general health on a 5‐point scale (excellent = 1 to bad = 5). We regressed endorsement (Y/N) of each risk factor on age, education, gender, and self‐rated health. We also summed endorsed risk factors and regressed this overall risk count on the four predictors.

**Result:**

Risk factors, from most to least endorsed, were age = 75.6%, physical inactivity = 34.4%; high blood pressure = 26.2%, smoking = 14.7%, diabetes = 14.0%, gender = 11.8%, and education = 5.4%. Overall risk count averaged M = 1.8 (range 0‐7) and self‐rated health averaged M = 2.2 (range 1‐4). Age, gender, and education did not predict endorsement of any risk factor, whereas lower self‐rated health predicted a higher likelihood of endorsing diabetes (OR = 1.6, CI 1.1 = 2.6) but not of any other risk factor. None of the predictors were associated with overall risk count. We examined the contribution of education further by including an education by age interaction term but found no significant relationships in these models either.

**Conclusion:**

We found that across educational groups, perceptions of personal risk for AD/dementia appear to be insufficiently grounded in knowledge. Our findings align with previous research highlighting the inadequacy of knowledge around AD/dementia risk factors and risk reduction among older adults. Future health education initiatives should specifically promote a nuanced understanding of risk factors.

